# Dr. John J. A. Burke

**Published:** 1910-09

**Authors:** 


Dr. John J. A. Burke, of Rochester, died from paralysis at Clifton Springs, N. Y., August 1, 1910, aged 60 years. He went to the latter place for treatment but failed to improve as hoped. He graduated in medicine at the University of Buffalo in 1887, and served for nine years as health officer of Rochester.

